# Supplementary material for: Factors associated with stroke recurrence and mortality in ischemic and haemorrhagic stroke: Protocol for a systematic review and meta-analysis
Source: PLoS One. 2025 Aug 14;20(8):e0329932. doi: 10.1371/journal.pone.0329932 (PMC12352634; doi:10.1371/journal.pone.0329932)
Supplement: S2 Table — (DOCX) [file pone.0329932.s002.docx]

## **S2 Table. PubMed Search strategy**

| "stroke"[MeSH Terms] OR "ischemic stroke"[MeSH Terms] OR "embolic stroke"[MeSH Terms] OR "thrombotic stroke"[MeSH Terms] OR "hemorrhagic stroke"[MeSH Terms] OR "stroke*"[Title/Abstract] OR "ischemic stroke*"[Title/Abstract] OR "embolic stroke*"[Title/Abstract] OR "thrombotic stroke*"[Title/Abstract] OR "hemorrhagic stroke*"[Title/Abstract] OR "haemorrhagic stroke*"[Title/Abstract] OR "cerebrovascular accident*"[Title/Abstract] OR "CVA"[Title/Abstract] OR "transient ischemic attack*"[Title/Abstract]   AND   "mortality"[MeSH Terms] OR mortality[Title/Abstract]   AND   "recurrence"[MeSH Terms] OR "recurrence*"[Title/Abstract] OR relapse* [Title/Abstract]    AND   "risk factors"[MeSH Terms] OR "risk factor*"[Title/Abstract] OR risk* [Title/Abstract] |
| --- |
