# Supplementary material for: Factors associated with stroke recurrence and mortality in ischemic and haemorrhagic stroke: Protocol for a systematic review and meta-analysis
Source: PLoS One. 2025 Aug 14;20(8):e0329932. doi: 10.1371/journal.pone.0329932 (PMC12352634; doi:10.1371/journal.pone.0329932)
Supplement: Attachment — Submitted filename: Response to Reviewers.docx [file pone.0329932.s004.docx]

| **Reviewer/editor comments** | **Author response** |
| --- | --- |
| *Reviewer #1:*  The authors present a well-designed systematic review protocol aimed at identifying clinical and lifestyle risk factors associated with recurrence of hemorrhagic and ischemic stroke. The justification for the future review is well-founded, and the authors have met all the requirements for a systematic review of observational studies. I congratulate the authors on the quality of the manuscript but suggest that they rethink selecting only articles published in English, since more and more technologies, artificial intelligence, and sites such as DeepL Translate make it possible to translate articles published in other languages into English with good quality. Therefore, in order to avoid this limitation put forward in the discussion, I suggest you review and consider including studies published in any language, with the explanation that they will be translated into English with good-quality free technologies available. | We thank Reviewer #1 for their time reviewing this manuscript and the feedback provided. We agree with the suggestion provided regarding considering non-English studies for inclusion and have decided to amend this in the protocol. Amendment below:  *‘No restrictions will be placed on publication language; the full-text of non-English studies retrieved will be translated to English using freely available, high-quality translation technologies, such as DeepL Translate, and then screened for eligibility.’* |
| *Editor:*  Please ensure that your manuscript meets PLOS ONE’s style requirements, including those for file naming. | We apologise for this oversight and have ensured that the manuscript now meets the style requirements. File names for supplementary material have been amended to meet style requirements |
| *Editor:*  Please include captions for supporting information files at the end of your manuscript, and update any in-text citations to match accordingly. | We apologise for this oversight and have amended this in the manuscript. Captions for supporting information files are now appended at the end of the manuscript and in-text citations have been updated. |
| *Editor:*  Please review your reference list to ensure that it is complete and correct. | We have reviewed the reference list and can confirm that it is complete and correct. |
